# Expression of LRIG1 is Associated With Good Prognosis for Human Non-small Cell Lung Cancer

**DOI:** 10.1097/MD.0000000000002081

**Published:** 2015-10-30

**Authors:** Yuzhi An, Zibo Zhao, Pengju Ou, Guangchuan Wang

**Affiliations:** From the Department of Oncology, the First Affiliated Hospital of Liaoning Medical University, Jinzhou, Liaoning, P.R. China (YA); Department of Oncology, University of Wisconsin-Madison, Madison, WI (ZZ); and Department of Immunology, Liaoning Medical University, Jinzhou, Liaoning, P.R. China (PO, GW).

## Abstract

Somatic mutations, which are associated with a certain rate of response to targeted therapies, are ubiquitously found in human non-small cell lung cancer (NSCLC). However, it is largely unknown which group of patients may benefit from the respective treatments targeting different somatic mutations. Therefore, more effective prognostic and predictive markers are desperately needed for the treatment of NSCLC harboring different somatic mutations. The leucine-rich repeats and immunoglobulin-like domains (LRIG)-1 is a tumor suppressor gene that belongs to the LRIG family. LRIG1 expression has prognostic significance in various human cancers.

In this study, we first used the quantitative polymerase chain reaction (qPCR) and immunohistochemical analysis of 36 and 182 NSCLC patient tissues to analyze the LRIG1 expression respectively. To investigate the prognostic value of LRIG1 in NSCLC, we examined the correlation between clinical features and overall survival (OS) with Cox proportional hazard regression. We also compared the sensitivity and specificity of LRIG1 in NSCLC prognosis by logistic regression to further evaluate the prognostic efficiency of LRIG1 in NSCLC.

We found that the LRIG1 expression was associated with pathological type, differentiation status, and stage of NSCLC. The result showed that LRIG1 was an independent prognostic factor for OS of NSCLC patients. LRIG1 in combination with other clinicopathological risk factors was a stronger prognostic model than clinicopathological risk factors alone.

Thus, the LRIG1 expression potentially offered a significant clinical value in directing personal treatment for NSCLC patients.

## INTRODUCTION

In the past several decades, platinum-based doublet regimens are the mainstay of chemotherapy in patients with advanced nonsmall-cell lung cancer (NSCLC).^1^ Nevertheless, somatic mutations in the epidermal growth factor receptor (EGFR), anaplastic lymphoma kinase (ALK), and K-Ras are widely found in NSCLC. These mutations are associated with a certain rate of response to targeted drugs, including erlotinib, gefitinib, and crizotinib.^2–4^ However, it remains elusive which group of patients benefit from the respective treatments. Therefore, more effective prognostic and predictive markers are desperately needed to predict the response to the targeted drugs in NSCLC patients.

The human leucine-rich repeats and immunoglobulin-like domains (LRIG) gene family is comprised of 3 genes including LRIG1, LRIG2, and LRIG3.^5–7^ Multiple studies have shown that LRIG1 may function as a tumor suppressor in human cancers.^8–10^ LRIG1 interacts with EGFR and enhances its ligand-stimulated ubiquitination and degradation.^11–13^ Therefore, LRIG1 negatively regulates EGFR and high expression of LRIG1 correlates with increased sensitivity to platinum-based and other cytostatic drugs in bladder cancer and esophageal carcinoma.^14–16^ Furthermore, high LRIG1 expression shows good prognosis and correlates with a longer disease-free survival and/or overall survival in squamous cell carcinoma of the skin,^4^ breast cancer,^17^ cervical cancer,^18^ and oropharyngeal cancer.^10^

Lung cancer is the most frequently diagnosed cancer and the leading cause of cancer-related deaths worldwide and is responsible for ∼13% of the total new cases and 18% of the deaths per year.^19–21^ The majority of this disease is diagnosed as NSCLC, which accounts for >80% of all cases of lung cancer^22^ and remains incurable when the cancer cells metastasize to the other organs. Currently, a limited number of studies have investigated the roles of LRIG1 in lung cancer. The LRIG1 protein has been shown to be expressed in normal human lung cells.^2^ LRIG1 expression was downregulated in certain tumor cell lines compared to the corresponding normal tissues.^23^

In the present study, we investigated the expression of LRIG1 mRNA levels by the quantitative polymerase chain reaction (qPCR) method and detected the LRIG1 protein level in the test and validation cohorts by immunohistochemistry (IHC). We found that the LRIG1 expression was associated with pathological type, differentiation status, and stage of NSCLC. For survival analyses, the Kaplan–Meier method was used to analyze the correlation between overall survival (OS) and variables. The log-rank test was used to compare survival curves. We analyzed the correlation between variables and OS using Cox proportional hazards regression, and receiver operating characteristic (ROC) curves were used to compare the prognostic accuracy of LRIG1 with clinicopathological risk factors in these NSCLC patients. The result showed that LRIG1 was an independent prognostic factor for OS of NSCLC patients.

## MATERIALS AND METHODS

### Patients and Clinical Characteristics

**FIGURE 1:**
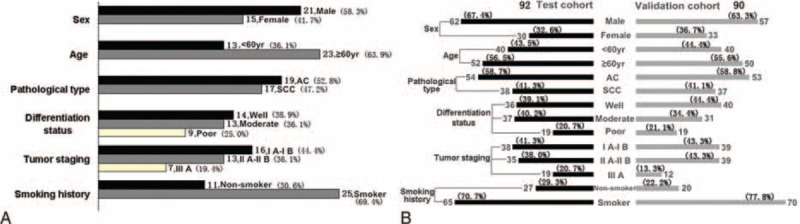
Clinical characteristics of the patients. (A,B) The strip represents the number of patients in different groups. (A) The clinical characteristics of 36 NSCLC fresh tissue samples for quantitative PCR. (B) The clinical characteristics of 182 formalin-fixed paraffin-embedded NSCLC tissue samples for immunohistochemistry. NSCLC = nonsmall cell lung cancer, PCR = polymerase chain reaction.

The patient samples and the study approval were discussed as previously described.^21^ In this study, 36 NSCLC fresh tissue samples and 182 formalin-fixed paraffin-embedded (FFPE) NSCLC tissue samples were obtained between December 2001 and April 2012 from Liaoning Medical University Affiliated First Hospital in China. The patients who had histories of other solid tumors, and/or had incomplete clinicopathological and follow-up data were excluded. The patients did not undergo radical surgery treatment, radiotherapy, chemotherapy, or other anticancer therapies prior to surgery. Moreover, all samples were randomly selected regardless of age, gender, or duration of the diseases, and all cases were diagnosed pathologically.^21^ All cases were classified according to the World Health Organization (WHO) revised proposal for histological types of lung cancer.^24^ The study was performed with respect to the ethical standards of the 1975 Declaration of Helsinki, as revised in 2000 and was approved by the Ethics Committee of Liaoning Medical University in China.^21^ Random numbers were used to assign 92 samples for test cohort and 90 samples for validation cohort. The follow-up's deadline was in September, 2014. OS was defined as the time from the date of surgery to the date of death or the last follow-up examination. The clinicopathological data was summarized in Figure 1.

### Quantitative Polymerase Chain Reaction (qPCR)

The qPCR reactions were performed as previously described.^21^ Briefly, total RNA was extracted using the TRIzol RNA kit (Invitrogen Life Technologies, Carlsbad, CA). The first-strand complementary DNA (cDNA) was prepared with the first-strand PrimeScript™ RT reagent kit containing the gDNA Eraser (Takara Bio, Inc, Shiga, Japan). Totally, 1 μg of RNA was used for the reverse transcription (RT) reaction. The RT reaction was performed under the conditions as previously described.^21^ The PCR was performed using primers specific for LRIG1 and the housekeeping gene, glyceraldehyde-3-phosphate dehydrogenase (GAPDH). The following primer sequences were used: LRIG1 sense, 5′-CCTGGAGTTGGGAGCATTTGA-3′ and antisense, 5′-CCGAATCCTGTTCCGATTGAG-3′ (PCR product length, 142 bp); and GAPDH sense, 5′-GCACCGTCAAGGCTGAG AAC-3′ and antisense 5′-TGGTGAAGACGCCAGTGGA-3′ (PCR product length, 138 bp).

The qPCR was run with a Mastercycler^®^ ep realplex (Eppendorf, Hamburg, Germany) using the SYBR^®^ Premix Ex Taq™ kit (Takara Bio, Inc) as previously described.^21^

### Immunohistochemistry (IHC)

IHC was performed on the formalin-fixed paraffin sections as previously described.^21^ Briefly, the tissue sections were processed to inactivate the endogenous peroxidase. Antigens were retrieved with 0.01 M sodium citrate buffer (pH 6.0) under high pressure for 2 min. Next, the sections were incubated with 2 μg/mL anti-LRIG1 primary antibody (Ab36707, Abcam, Cambridge, UK) at 4°C overnight and then immunostained with a horseradish peroxidase/Fab polymer-conjugated secondary antibody (Beijing Zhongshan Golden Bridge Biotechnology Co, Ltd, Beijing, China) for 30 min at room temperature.^21^ The signal was revealed by diaminobenzidine at room temperature for 1 min and counterstained with hematoxylin for another 15 min. Two independent investigators who were blinded to the clinical details examined and scored all sections, and at least 5 fields were randomly selected.^21,25^ The expression was scored as “high” when ≥50% of the cancer cells were immunopositive and as “low” when <50% of the cancer cells were immunopositive or negative.^21^ This cut-off criterion was selected as it showed the best explanatory power of the various cut-offs tested (0, 20, 50, and 100%).^21^

### Statistical Analysis

The statistical analysis was performed as previously described.^26^ SPSS 18.0 software (Chicago, IL) was used. Two-sample test for independent samples and χ^2^ test was used for continuous and categorical variables, respectively. To compare the prognostic accuracy of LRIG1 with clinicopathological risk factors in all patients, we used ROC curves. To analyze the correlation between OS and variables, the Kaplan–Meier method was applied for survival analyses. The log-rank test was carried out to compare survival curves. Multivariate analysis was carried out with the Cox proportional hazard regression model with stepwise manner (forward: LR, entry α=0.05, stay α=0.1). Statistical significance was set at *P* ≤ 0.05.

## RESULTS

### Patients and Clinical Characteristics

In this study, 36 NSCLC fresh tissue samples and 182 formalin-fixed paraffin-embedded (FFPE) NSCLC tissue samples were obtained. Random numbers were used to assign the 92 samples for test cohort and the 90 samples for validation cohort. The clinicopathological data was summarized in Figure 1.

### Analyzes mRNA Expression of LRIG1: Correlation With Clinical Characteristics

The melting curve and the gel electrophoresis analysis demonstrated specific target and reference gene amplification. The slopes of the standard curves were calculated to be −3.242 and −3.246 for the GAPDH and LRIG1 genes, respectively. We then assessed the reliability of the PCR reaction efficiencies by plotting ΔCT values defined as “C_T_LRIG1-C_T_GAPDH.” The absolute value of the trend line slopes was ≤0.1. This result indicated the validity of the relative quantitative assay by the ΔΔCT method.^21^

**FIGURE 2:**
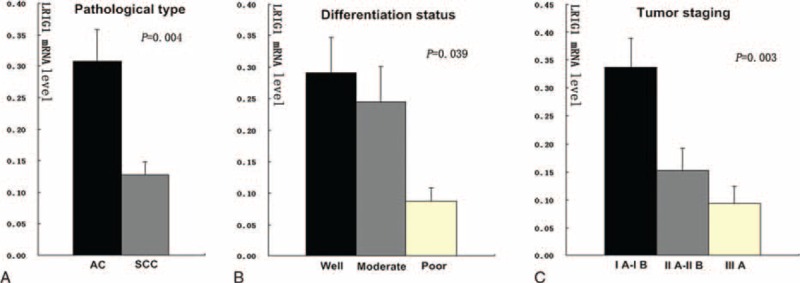
The expression of LRIG1 in NSCLC by quantitative PCR. (A) The expression of LRIG1 in various pathological type (*P* = 0.004); (B) the expression of LRIG1 in various differentiation status (*P* = 0.039); (C) the expression of LRIG1 in various tumor stages (*P* = 0.003). Y-axis represents the expression of LRIG1 mRNA level. LRIG1 = leucine-rich repeats and immunoglobulin-like domains-1, NSCLC = non-small cell lung cancer, PCR = polymerase chain reaction.

The LRIG1 mRNA levels were examined in 36 NSCLC tissues using the ΔΔCT method. The correlation between LRIG1 mRNA levels and clinical characteristics in the cancer tissues (pathological type, differentiation status, and tumor staging) was further analyzed (Fig. 2). The expression of LRIG1 mRNA was significantly higher in adenocarcinoma (AC) compared with that in squamous cell carcinoma (SCC) (*P* = 0.004) (Fig. 2A). A significant downregulation of LRIG1 (*P* = 0.039) was also observed in the tumors that were poorly differentiated (Fig. 2B). Notably, a significant correlation was observed between the LRIG1 expression and the tumor stage (*P* = 0.003) (Fig. 2C). Nevertheless, no correlation was observed between LRIG1 mRNA levels and age, sex, or smoking history of these patients.

### Correlation Between LRIG1 Protein Expression and Clinical Characteristics of NSCLC

**FIGURE 3:**
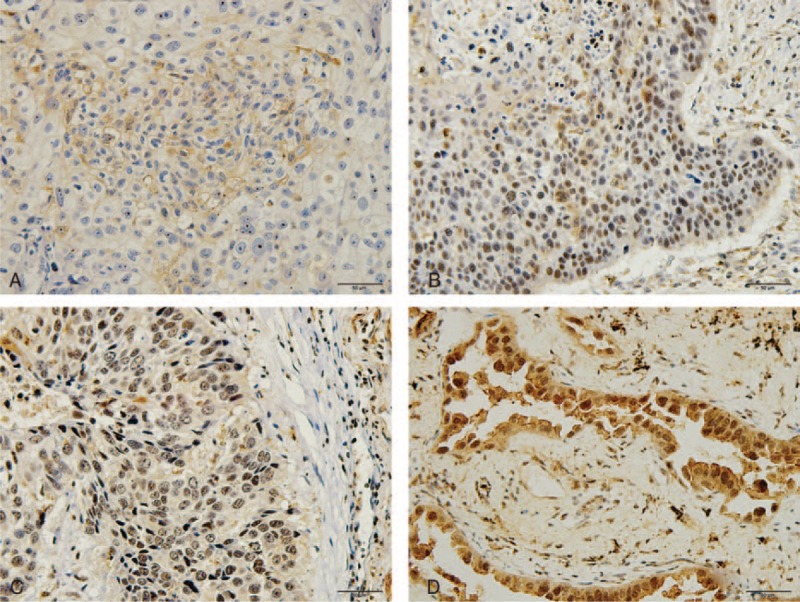
Immunostaining for LRIG1 in NSCLC tissues. The LRIG1 protein was mainly expressed in the nucleus of (A) negative, (B) mild, (C) moderate, (D) high expressions with brownish yellow staining. LRIG1 = leucine-rich repeats and immunoglobulin-like domains-1, NSCLC = non-small cell lung cancer.

To evaluate the protein expression of LRIG1 in NSCLC, 92 (test cohort) tissue samples and 90 (validation cohort) samples were detected by immunohistochemistry (Fig. 3). About 48 patients (52.17%) in the test cohort and 54 patients (60.00%) in the validation cohort were classified into the high-LRIG1 group. Statistical analysis demonstrated that the LRIG1 protein levels were highly correlated with the clinical characteristics including pathological type, differentiation status, and tumor stage, but not with sex or age in both cohorts. This result was also consistent with the qPCR result (Fig. 2). Moreover, the percentage of high-LRIG1 population was higher in the nonsmoker group in the validation cohort as shown in Table 1.

**TABLE 1:**
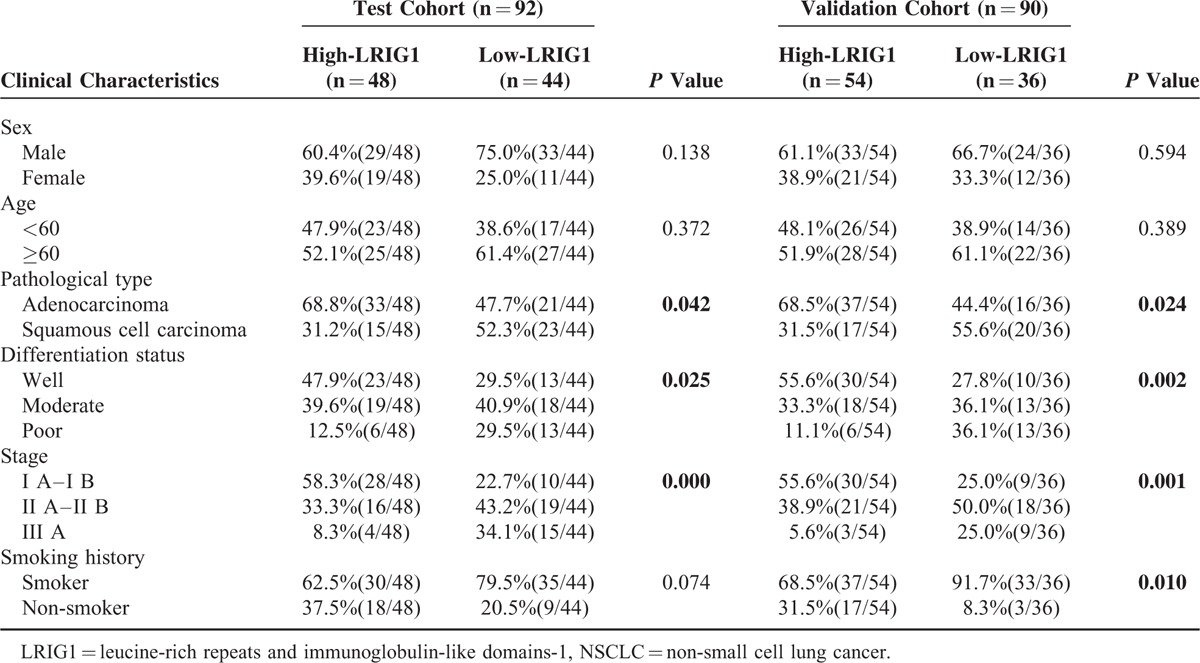
Relationship Between LRIG1 Expression and Clinical Characteristics of NSCLC in Test and Validation Cohorts

### LRIG1 is an Independent Prognostic Factor for the OS of NSCLC Patients

**FIGURE 4:**
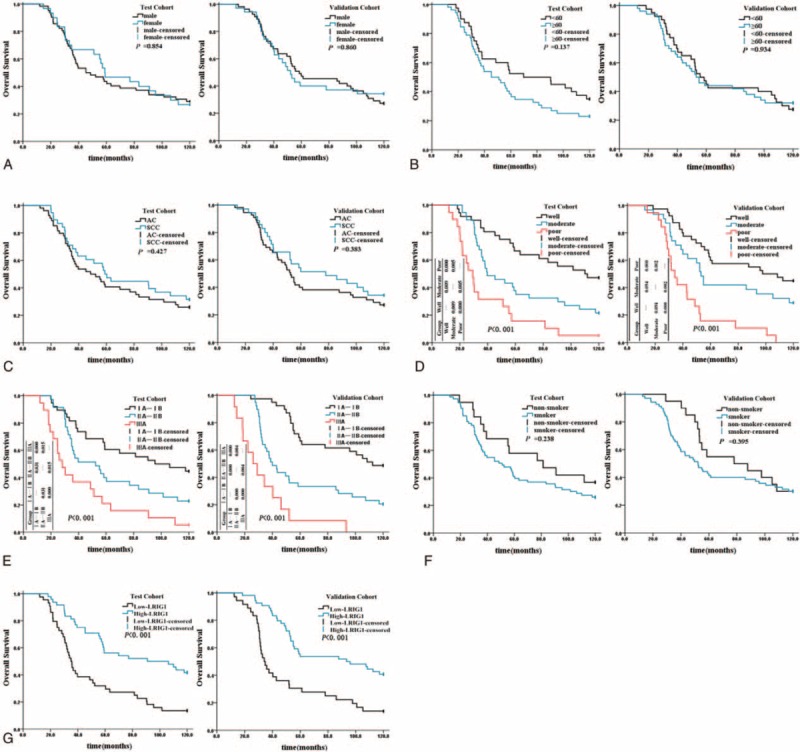
Kaplan–Meier survival curves of relationship between clinical features and OS in NSCLC patients. (A) The overall survival of NSCLC patients with different sex was not significance in both cohorts (*P* = 0.854, *P* = 0.860); (B) the overall survival of NSCLC patients with different age was not significant in both cohorts (*P* = 0.137, *P* = 0.934); (C) the overall survival of NSCLC patients with different pathological types was not significant in both cohorts (*P* = 0.427, *P* = 0.383); (D) the overall survival of NSCLC patients with various differentiation status was significant in both cohorts (*P*<0.001, *P*<0.001); (E) the overall survival of NSCLC patients with various tumor staging was significant in both cohorts (*P*<0.001, *P*<0.001); (F) the overall survival of NSCLC patients with different smoking history was not significance in both cohorts (*P* = 0.238, *P* = 0.395); (G) the overall survival of NSCLC patients with different LRIG1 was significant in both cohorts (*P*<0.001, *P*<0.001). NSCLC = non-small cell lung cancer, OS = overall survival.

**FIGURE 5:**
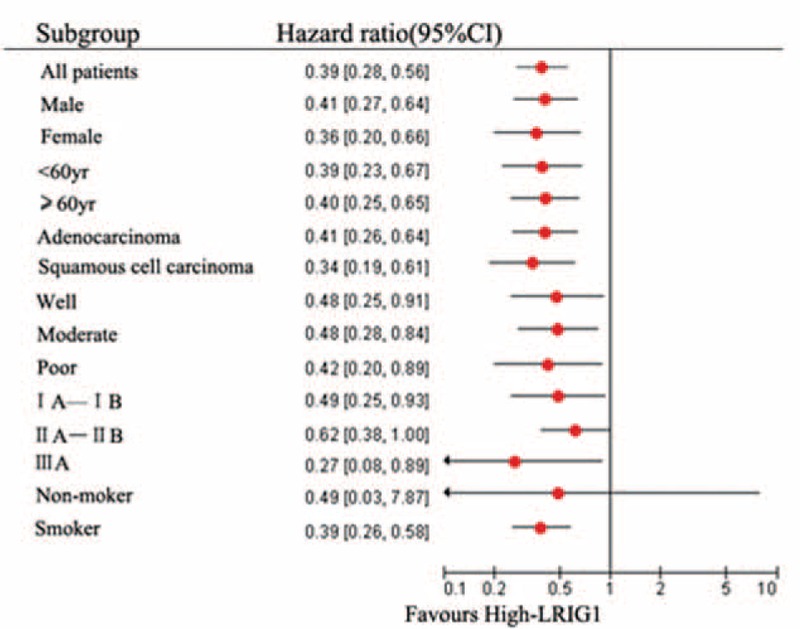
Forest plot of the relationship between the expression of LRIG1 in various clinical features and mortality risk in NSCLC patients. The red dots represent the average location. Overall survival of high-LRIG1 group was significantly better than that of the low-LRIG1 group in all subgroups except the IIA–IIB group and the nonsmoker group. LRIG1 = leucine-rich repeats and immunoglobulin-like domains-1, NSCLC = non-small cell lung cancer.

In the follow-up survey of 182 cases of NSCLC patients, the median survival time was 55.3 months. The prognostic value of LRIG1 for the OS in NSCLC patients was evaluated using the Kaplan–Meier method. We also compared the survival curves using the log-rank test in our test and validation cohorts. Our results showed that the OS of the high-LRIG1 group was significantly higher than that of the low-LRIG1 group in both cohorts (Fig. 4G). The differentiation status, tumor stage, and LRIG1 expression levels were predictors of OS in the 2 cohorts as demonstrated by the univariate analysis (Fig. 4). In order to define the role of LRIG1 protein in the subgroup for all 182 cases, the forest plot was adopted. The result showed that the OS of high-LRIG1 group was significantly higher than that of the low-LRIG1 group in all subgroups except the IIA–IIB group and the nonsmoker group (Fig. 5). Furthermore, multivariate Cox proportional hazards regression analysis indicated that the differentiation status, tumor stage, and LRIG1 expression levels were independent prognostic factors for NSCLC in both cohorts (Table 2).

**TABLE 2:**
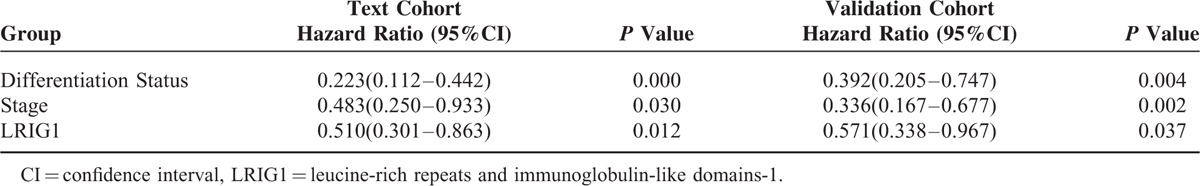
Multivariate Cox Proportional Hazards Regression Analysis

**FIGURE 6:**
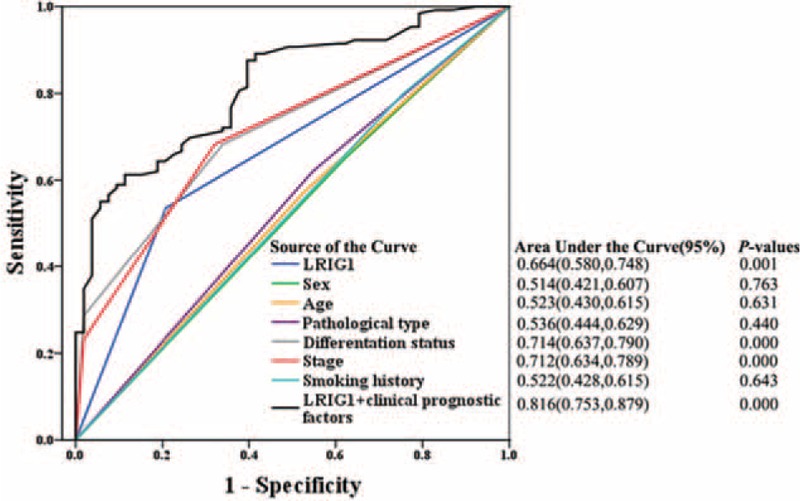
The ROC curve of 8 models (clinical characteristics, LRIG1, and LRIG1 combined with clinical characteristics). The area under the curve was 0.664 for LRIG1 (*P* = 0.001) and 0.816 for LRIG1 combined with clinical characteristics (*P*<0.001). LRIG1 = leucine-rich repeats and immunoglobulin-like domains-1, ROC = receiver operating characteristic.

Next, we used ROC curves to compare the sensitivity and specificity of LRIG1 in predicting NSCLC prognosis in order to further confirm the prognostic accuracy of LRIG1.^26^ The variables were pretreated by logistic regression before the analysis. Eight models were analyzed including LRIG1 in combination with multiple clinical characteristics (Fig. 6). The area under the curve (AUC) was 0.664 for LRIG1, 0.714 for differentiation status, 0.712 for stage, 0.816 for LRIG1 combined with all clinical characteristics. Our results revealed that AUC for LRIG1 combined with these clinical characteristics prognostic factors was significantly higher than any of the individual factors (Fig. 6).

## DISCUSSION

Epidermal growth factor receptor (EGFR) overexpression and chromosome 3p deletion are 2 frequent events in lung cancers. LRIG1, a negative regulator of EGFR, was found at 3p14^23,27^ and its copy number loss correlated with poor clinical outcome.^4,10,17,18^ The gene is the human homolog of mouse Lrig1^28^ and encodes a transmembrane protein which, in the extracellular part, shows structural similarities to the drosophila cell surface protein Kekkon-1.^29^ The extracellular part of Kekkon-1 functions in drosophila as an inhibitor of EGFR-mediated signaling.^30^ As far as we know, the case is similar in humans, though more complicated.

In this study, according to tumor differentiation status and stages, a significant downregulation of LRIG1 mRNA was observed in NSCLC tissues (Fig. 2B and C). In other words, lower LRIG1 mRNA levels can result from poor differentiation status and advanced stage of NSCLC, a type of tumors with worse prognosis. Interestingly, the expression of LRIG1 mRNA was significantly higher in AC compared with that in SCC (Fig. 2A). Therefore, LRIG1 in different pathological types may have differential effects, which warrants further investigation.

In the following study, 182 cases were randomly assigned to 92 samples (test cohort) and 90 samples (validation cohort) and were detected by immunohistochemical methods (Fig. 3), which further reinforced the results of our qPCR data (Fig. 2). Previously, Lindstrom AK et al found that women who lacked LRIG1 expression had a significantly increased smoking frequency.^31^ In validation cohort, we found that there was a significantly difference in the smoking history according to the LRIG1 expression, and more high-LRIG1 cases were observed in the nonsmokers (Table 1).

In this study, we found that the staining pattern of LRIG1 was predominantly nuclear in both subgroups (AS and SCC) by IHC (Fig. 3). Still, a small amount of expression could be observed in the cytoplasmic compartment (Fig. 3). Based on the previous studies, LRIG proteins showed differential subcellular localizations depending on the specific cell types.^8,10^ For example, in astrocytic tumors, LRIG1 staining can be found in the nuclear, perinuclear, and cytoplasmic compartments, and only the perinuclear staining correlates with low WHO grade.^32^ In the normal skin cells, nuclear LRIG1 expression is predominant; however, in the skin cells of psoriatic patients, LRIG1 is redistributed from cell nuclei to the cytoplasm.^33^ Therefore, there is a differential subcellular distribution of LRIG1 proteins in different cell types, and the role of the nuclear LRIG1 staining pattern found in NSCLC remains unknown and warrants further investigation.

To confirm the significant value of LRIG1 for predicting the prognosis in NSCLC, we have analyzed the correlation between sex, age, pathological type, differentiation status, tumor staging, smoking history and LRIG1 expression levels with OS (Fig. 4), and assessed the prognostic efficacy of LRIG1. The results demonstrated that differentiation status, tumor staging, and LRIG1 expression levels were all independent prognostic factors for NSCLC. In the ROC curve analysis, AUC of LRIG1 was 0.664. This further proved its prognostic value albeit with low accuracy. However, considering the influence of different pathological types and smoking history, a further single pathological types and smoking history analysis are desired to improve the prognostic value of LRIG1.

High expression of LRIG1 correlates with better patient survival in multivariate analyses where other known risk factors were included.^4,10,17,18^ Studies have demonstrated that the LRIG1 expression is associated with human papillomavirus (HPV) status.^10^ LRIG1 is an independent positive prognostic marker patients with HPV-positive tumor.^10^ In the II A–II B and nonsmoker groups, LRIG1 expression did not correlate with patient's overall survival (Fig. 5). These results showed that the evaluation of LRIG1 expression in NSCLC may provide further prognostic information in addition to the previously known risk factors.

Various methods have been used to evaluate the LRIG1 expression in different literatures.^18,34,35^ The study reported by Kvarnbrink et al analyzed both protein and mRNA levels of LRIG1 expression in 2 different cohorts.^35^ A 0 to 3 scoring system was used for analyzing the IHC staining intensities and LRIG1 mRNA levels were stratified as low, intermediate, and high groups. The study reported by Muller et al analyzed the immunoreactivity of LRIG1-3 in 86 cervical adenocarcinoma cases.^18^ The percentage of positive cells was based on a 5-grade semiquantitative scale. The study reported by Ghasimi et al analyzed the immunoreactivity of LRIG proteins in 409 meningiomas and the correlation with estrogen receptor status as well.^34^ The cytoplasmic and nuclear immunoreactivities were scored with different cut-off values. In our study, the expression was scored as high when ≥50% of the cancer cells were immunopositive and as low when < 50% of the cancer cells were immunopositive or negative. A standardized histochemical scoring should be developed to generate data with high consistency in the future studies.

A recent study showed that LRIG1 is a prognostic biomarker in nonsmall cell lung cancer.^35^ In their study, a relatively large number of cases of NSCLC were collected from an established tissue microarray (TMA) and the Oncomine database. The association between the LRIG1 mRNA level and patient survival was analyzed by retrieving the data from the Oncomine database. The samples we collected come from different regions and even different countries. According to numerous studies, the expression pattern of human genes is very likely to show regional differences. Hence, it is crucial to study the same factors from different regions. The repeatability of the experiments was improved by using the method of self-contrast. From different perspectives, our experimental conclusion is more comprehensive. We have analyzed the correlation between variables and overall survival using Cox proportional hazards regression, and ROC curves were used to compare the prognostic accuracy of LRIG1 with clinicopathological risk factors in NSCLC patients. We believe these differences make our study important and significant as well.

Losing follow-up is one of the reasons for the biases in the observational study, and at the time of follow-up, the patient or family members of the diseases and the basic situation of the statement was not clear which may result in the experimental results biases in the observational study. However, in conclusion, LRIG1 was found to be an independent positive prognostic marker in NSCLC. In patients with different pathological type, differentiation status, tumor staging, the evaluation of LRIG1 may offer additional prognostic information. This study provides important insight into the prognostic value of LRIG1 in NSCLC and the functional role of the LRIG1 in NSCLC warrants further investigation.
